# “Are we forgetting the smallest, sub 10 nm combustion generated particles?” 

**DOI:** 10.1186/s12989-015-0107-3

**Published:** 2015-10-31

**Authors:** Paola Pedata, Tobias Stoeger, Ralf Zimmermann, Annette Peters, Günter Oberdörster, Andrea D’Anna

**Affiliations:** Department of Experimental Medicine - Section of Hygiene, Occupational Medicine and Forensic Medicine – School of Medicine, Second University of Naples, Via L. De Crecchio 7, 80138 Naples, Italy; Comprehensive Pneumology Center, Institute of Lung Biology and Disease (iLBD), Helmholtz Zentrum München, Ingolstädter Landstraße 1, D-85764 Neuherberg, Germany; Comprehensive Molecular Analytics/Joint Mass Spectrometry Centre, Helmholtz Zentrum München, Ingolstädter Landstraße 1, 85764 Neuherberg, Germany; Analytical Chemistry/Joint Mass Spectrometry Centre, Institute of Chemistry, University of Rostock, Dr-Lorenzweg 1, 18051 Rostock, Germany; Institut of Epidemiology II, Helmholtz Zentrum München, Ingolstädter Landstraße 1, 85764 Neuherberg, Germany; Department of Environmental Medicine, Emeritus of Toxicology, University of Rochester, Rochester, 14642 NY USA; Department of Chemical, Material and Industrial Production Engineering, University of Naples “Federico II”, P. Tecchio 80, 80125 Naples, Italy

**Keywords:** Nanoparticles, Combustion-generated particles, Surface area, Diesel filter, Toxicity, Ultrafine particles

## Abstract

Although mass emissions of combustion-generated particulate matter have been substantially reduced by new combustion technology, there is still a great concern about the emissions of huge numbers of sub-10 nm particles with insignificant mass. These particles have up to orders of magnitude higher surface area to mass ratios compared to larger particles, have surfaces covered with adsorbed volatile and semi-volatile organic species or even are constituted by such species. Currently there is only very little information available on exposure and related health effects specific for smaller particles and first evidences for long-term health effects has only been recently published. However, the fact that these nanoparticles are not easily measured at the exhausts and in the atmosphere and that their biological activity is obscure does not mean that we can overlook them. There is an urgent need to develop i) reliable methods to measure sub-10 nm particles at the exhaust and in the atmosphere and ii) a robust correlation between the chemical structure of the molecules making up combustion-generated nanoparticles and health burden of new combustion technologies. Our attention has to turn to this new class of combustion-generated nanoparticles, which might be the future major constituents of air pollution.

## Background

Fossil fuel burning emits particulate matter when incomplete combustion caused by locally fuel-rich conditions generates high-molecular-mass aromatic compounds from fuel carbon. The size distribution function of combustion-generated particles is generally bimodal [1, 2]. It has a nucleation mode, sizes between 2 and 10 nm, mostly constituted of organic carbon, and an accumulation mode, larger sizes composed of more elemental carbon dominated soot particles [3].

Nucleation mode particles are formed in local slightly fuel-rich conditions, when the mixing at atomic level is poor; they are believed to be stacks of few aromatic molecules held together by van der Waals interactions and they are soot precursor particles, when operating conditions allow soot to be formed. Combustion systems with high levels of turbulent mixing of fuel and oxidant, such as new-technology engines, and processes characterized by bluish flame luminosity, are prone to form nanoparticles and not soot [1, 4].

Combustion-generated particles easily can adsorb molecules from the gas phase, such as NOx, benzene and Polycyclic Aromatic Hydrocarbons or reactive compounds upon cooling of the exhaust. Furthermore, these latter potentially hazardous compounds are prone to form condensation particles and organic aerosol in the atmosphere with sizes comparable with nucleation mode particles [5].

Combustion technologists have developed a combination of advanced exhaust control systems, and have reformulated fuels to reduce particulate matter emission. Noteworthy, for new-technology diesel engines mass emissions have been substantially reduced and a recent chronic rat inhalation study revealed no significant biologic responses, as compared to emissions of traditional-technology engines [6].

**Fig. 1:**
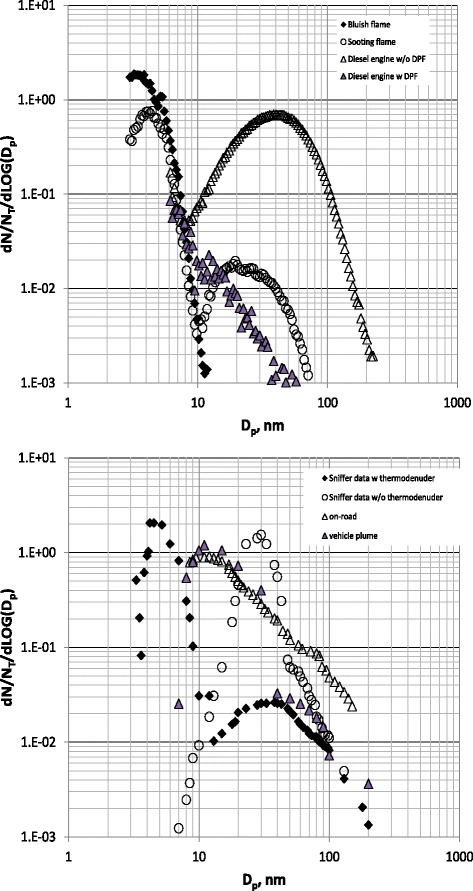
Upper panel: Normalized particle size distribution functions measured in the bluish (full diamond) and in the sooting region of an ethylene-rich/air flame (empty circle), and at the exhaust of a common-rail Diesel engine at half load with (grey triangle) and without (line) a particulate filter (DPF). Flame data adapted from Echavarria et al. [26]. Engine data adapted from D’Anna A. [1] & Gualtieri M. et al. [18]. Lower panel: Normalized particle size distribution functions measured on-road (empty triangles) and in vehicles plumes (gray triangles) or following the vehicle with a “sniffer” laboratory vehicle with (full diamonds) and without (empty circles) a thermodenuder. On-road data adapted from Kittelson et al. [22]; vehicle plumes adapted from Yao et al. [23]; “sniffer” data adapted from Ronkko et al. [4]

The main concern about combustion-generated particles arises because of the emissions in the atmosphere of huge numbers of sub-10 nm particles with insignificant mass. Figure 1 illustrates some representative normalized particle size distributions measured at the exhaust of combustion systems, including laminar flames and Diesel engine exhausts, with and without a particulate filter (upper panel).

Sub-10 nm particles have up to orders of magnitude higher surface area to mass ratios compared to larger particles, have surfaces covered with adsorbed volatile and semi-volatile organic species or even are constituted by such semi-volatile species when the sizes is in the order of 1–2 nm. In this context it is important to note that particle surface area is in general thought to scale best with the surface reactivity, bioactivity and toxicity of poorly soluble particles [7, 8] and that the organic content is often an additional major driver of the pulmonary inflammatory response [9]. Once inhaled, nanoparticles can deposit inside the airways and access the nasal, bronchial and the particularly fragile, alveolar epithelium [10]. Solid nanoparticles distinctly smaller than 100 nm translocate into and in between cells, reach the bloodstream and can be found in organs other than the lung [11]. Hence, in addition to the adverse effects in the lung and indirect effects on the cardiovascular system [12], nanoparticles potentially affect other organs such as the liver and the brain and may contribute to diseases beyond the lung and heart [13]. Thus, the health effect of sub-10 nm particles might go beyond what may be expected from their very low mass concentrations. Once deposited on the respiratory surface, the physicochemical nature of the sub-10 nm organic carbon particles will determine their toxicokinetic fate, but at present, the physicochemical characteristics of the sub-10 nm particles are poorly known and controversial, and strongly depend on the operating conditions in which particles are generated.

Combustion-generated particulate matter has been associated with respiratory and cardiovascular morbidity and mortality [12–15]. Currently there is however only very little information available on exposure and related health effects specific for smaller particles and first evidences for long-term health effects has only been recently published [16]. Nanoparticles formed in bluish laboratory flames as well as those emitted by 2004-model Diesel and gasoline vehicles have been shown to effectively interact with both prokaryotic and eukaryotic cells [17]. In vitro they produce a dose-dependent mutagenic response in Salmonella bacteria and above a critical dose, a significant cytotoxic response in mouse embryonic fibroblasts. First in vitro results indicate that in comparison to flame-formed nanoparticles - even if characterized by approximately the same sizes - engine emitted nanoparticles may bear a mutagenic potential. Since most of the molecules forming the nanostructure in these nanoparticles are actually at the surface, the biological reactivity seems strictly associated with molecular constituents as well as particle size. In this context, nanoparticles emitted from a diesel engine fueled with a diesel oil doped with additives, including aromatic and oxygenated components, induce cytotoxic and pro-inflammatory effects on epithelial cells which exceed the cytotoxic potential of particles produced from commercial fuel oil by ten times [18]. In another study, emissions from a diesel car equipped with a diesel particle filter induce pro-inflammatory effects in an air-liquid interface exposed three-dimensional model of the human airway epithelium [19]. Nanoparticles generated in a premixed flame showed a significantly stronger apoptotic response than that experienced by engineered carbonaceous nanoparticles of the same size [20, 21]. By contrast to these observations, nanoparticles generated by a domestic cooktop burner, fed with network natural gas, showed neither after a treatment of up to three days a reduction in epithelial cell viability, or any activation of pro-inflammatory pathways. The latter result highlights the impact of the chemical nature of the molecules forming the nanostructure, in addition to the nanoparticle size.

So far we are limited to view in vitro studies, but these preliminary results indicate that the emissions from bluish flames and from new-technology engines, at present considered relatively clean, are not yet sufficiently studied. The reduction of particulate mass emission rate supposedly not automatically lead to a reduction in toxic effects and a substantial improve in air quality as the emission of huge amounts of nanoparticles of insignificant mass may increase the biological, cytotoxic and inflammatory potential of these aerosols.

Today there is not yet a clear evidence of the presence of combustion-generated sub-10 nm particles in places close to emission sites such as traffic roads, highways or industrial areas. Indeed, the number concentration of particles with sizes below 10 nm decreases in on road measurements [22, 23] although bi-modal size distribution functions, very similar to those measured at the exhaust of lab engines and flames, are detected in vehicle plumes [4] (lower panel of Fig. 1). Differences between on road and plume measurements can be ascribed to different factors including condensation of volatile material on nonvolatile core particles in the atmosphere, fast coagulation of nanoparticles when released in the atmosphere [24], inadequacy of the sampling and measuring systems. There is however, the need for further validation studies, down to very low particle sizes, to assess the importance of sub-10 nm particles to air quality and health.

## Conclusion

Sub-10 nm nanoparticles are formed by new technologies combustion systems and largely emitted into the atmosphere. The fact that these nanoparticles are not easily measured at the exhausts and in the atmosphere and that their biological activity is uncertain does not mean that we can overlook them. We shall find a reliable method to measure sub-10 nm particles at the exhaust and in the atmosphere and a robust correlation between the chemical structure of the molecules making up combustion-generated nanoparticles in the environmental and potential health burden of new combustion technologies. Furthermore, the fundamental properties of these particles, such as their capacity to transport toxic volatile and semi-volatile combustion by-products in vulnerable region of the lung, need to be studied. New, sensitive approaches to study the health effects in vivo and in vitro by deep molecular-biological profiling of cellular effects (multi-omics approach) of cell and animal models exposed to combustion aerosols at the air-liquid interface may be helpful to address the biological effects of these emissions [25]. Therefore, there is an urgent need to establish the role of exposure measures capturing the complex properties of combustion nanoparticles and determine the health and biological effects in addition to PM_2.5_. Our attention has to turn to this new class of combustion-generated nanoparticles, which might be the future major constituents of air pollution.

## Abbreviations

DPF: Particulate filter
